# Structural variation and its potential impact on genome instability: Novel discoveries in the *EGFR* landscape by long-read sequencing

**DOI:** 10.1371/journal.pone.0226340

**Published:** 2020-01-15

**Authors:** George W. Cook, Michael G. Benton, Wallace Akerley, George F. Mayhew, Cynthia Moehlenkamp, Denise Raterman, Daniel L. Burgess, William J. Rowell, Christine Lambert, Kevin Eng, Jenny Gu, Primo Baybayan, John T. Fussell, Heath D. Herbold, John M. O’Shea, Thomas K. Varghese, Lyska L. Emerson

**Affiliations:** 1 Sentry Genomics, Baton Rouge, LA, United States of America; 2 Department of Chemical Engineering, Louisiana State University, Baton Rouge, LA, United States of America; 3 Huntsman Cancer Institute, University of Utah School of Medicine, Department of Oncological Sciences, Salt Lake City, UT, United States of America; 4 Roche Sequencing Solutions, Madison, WI, United States of America; 5 Pacific Biosciences, Menlo Park, CA, United States of America; 6 Huntsman Cancer Institute, Biorepository Molecular Pathology, Salt Lake City, UT, United States of America; 7 Huntsman Cancer Institute, University of Utah School of Medicine, Department of Surgery, Division of Thoracic Surgery, Salt Lake City, UT, United States of America; 8 Huntsman Cancer Institute, University of Utah School of Medicine, Department of Pathology, Salt Lake City, UT, United States of America; CNR, ITALY

## Abstract

Structural variation (SV) is typically defined as variation within the human genome that exceeds 50 base pairs (bp). SV may be copy number neutral or it may involve duplications, deletions, and complex rearrangements. Recent studies have shown SV to be associated with many human diseases. However, studies of SV have been challenging due to technological constraints. With the advent of third generation (long-read) sequencing technology, exploration of longer stretches of DNA not easily examined previously has been made possible. In the present study, we utilized third generation (long-read) sequencing techniques to examine SV in the *EGFR* landscape of four haplotypes derived from two human samples. We analyzed the *EGFR* gene and its landscape (+/- 500,000 base pairs) using this approach and were able to identify a region of non-coding DNA with over 90% similarity to the most common activating *EGFR* mutation in non-small cell lung cancer. Based on previously published *Alu*-element genome instability algorithms, we propose a molecular mechanism to explain how this non-coding region of DNA may be interacting with and impacting the stability of the *EGFR* gene and potentially generating this cancer-driver gene. By these techniques, we were also able to identify previously hidden structural variation in the four haplotypes and in the human reference genome (hg38). We applied previously published algorithms to compare the relative stabilities of these five different *EGFR* gene landscape haplotypes to estimate their relative potentials to generate the *EGFR* exon 19, 15 bp canonical deletion. To our knowledge, the present study is the first to use the differences in genomic architecture between targeted cancer-linked phased haplotypes to estimate their relative potentials to form a common cancer-linked driver mutation.

## Introduction

Over the past decade, the ability to examine the human genome beyond short segments of a few hundred base pairs (bp) has greatly improved with the advent of third-generation (i.e., long-read) sequencing technologies which have improved detection and characterization of structural variants. Although short-read technology has been of tremendous benefit in identifying many of the cancer-driver genes, it has significant limitations when it comes to identifying structural variation (SV). Short-read sequencing is often challenging for accurate calling of large structural variants, especially in highly repetitive regions of a genome [1–4]. SV occurs most commonly in the non-coding regions of the genome [5, 6]. Examples of SV are copy-number variants (CNV), such as deletions and duplications, and copy number neutral variants, such as inversions, translocations, and complex rearrangements [7–9]. SV within the human genome exceeds the variation imparted by single nucleotide variants (SNVs) and is the major contributor to DNA sequence variation among humans [10]. Although SNVs constitute the majority of variants in the human genome, SV affects far more bases. For example, the typical genome contains an estimated 2,100 to 2,500 structural variants, affecting ~20 million bases of sequence compared to the only ~4–5 million bases affected by SNVs [2, 3]. Some studies put the estimates of bases affected by SV much higher, between 50 million to 130 million [3]. The frequency of structural variants (≥ 50 bp) and small indel variants (<50 bp) has been shown to exceed 840,000 per diploid human genome. Thus, the average combined indel and structural variant incidence within the human genome is over 100 per megabase, and human structural variants outnumber protein coding genes by a factor of 20 [1, 3, 11]. Despite the extent of SV in the human genome, it does not appear to occur randomly. The 1000 Genomes Consortium studied 2,504 individuals and identified 3,163 specific regions of the genome (~13 percent of the genome) in which there were consistently three or more instances of SV [12].

Several models of ectopic structure-driven DNA damage are described in the literature as being associated with repetitive DNA [13, 14]. Repetitive sequences such as long interspersed elements (LINE) and SINE-VNTR-*Alu* elements (SVA) have been shown to mediate human SV [15–17]. Another type of repetitive DNA is microsatellites. Mutations in microsatellites were first associated with human neurodegenerative diseases, and several molecular mechanisms for their instability in this setting have been well described [18–21]. Mutations in microsatellites have also been linked to cancer [22, 23].

The most frequent repetitive sequence in the human genome is the *Alu* element. Human DNA contains over one million *Alu* elements that collectively account for over ten percent of the genome [24]. *Alu* elements have been found at many SV breakpoints [25–27]. *Alu* elements have also been linked to cancer [28–30]. Hundreds of deletions generated by *Alu-Alu* recombination events have been shown to be present in humans [31, 32]. Reports of *Alu* element conversion events are consistent with the view that *Alu-Alu* interactions can catalyze SV [33, 34]. It has also been reported that interactions may occur between *Alu* elements separated by up to 421,000 bp [35]. The presence of increased sequence homology among neighboring *Alu* elements provides further support for the view that interactions between *Alu* elements are not uncommon [36, 37].

The structural motif of inverted repetitive DNA has long been recognized as a source of human genomic instability [38, 39]. Long inverted repeats have recently been correlated with SV in cancer [40, 41] and fused inverted *Alu* element pairs have also been identified in cancer [29, 41]. *Alu* elements are primate specific repeats which generate the most common form of inverted repetitive DNA in the human genome. In recombinant yeast studies, inverted human *Alu* elements are measurably unstable when separated by up to 100 bp [42]. Inverted *Alu* element instability is therefore of increasing significance as recent studies have linked inverted repeats to SV in cancer [40, 41]. Cook *et al*. utilized this principle of prevalence and instability of inverted *Alu* elements to undertake a genome-wide examination of the human genome to identify distribution patterns of inverted vs direct-oriented *Alu* elements. This work revealed a statistically significant imbalance (depletion) of inverted *Alu* pairs when compared to direct-oriented *Alu* pairs [35]. This imbalance occurs within 421,000 bp windows flanking each *Alu* element. While there are slightly over one million human *Alu* elements, the number of *Alu* pairs within this imbalance window exceed 100 million. Because of the large population of *Alu* pairs within this ± 421,000 bp window, imbalances between inverted and direct-oriented *Alu* element pairs can be accurately measured within ± 0.5 percent statistical confidence (p<0.05). This finding of measurable imbalances in *Alu* pairs allowed for the development of a predictive computational model of human genome instability. This model was applied to 100 human genes and successfully predicted the deletion-prone cancer genes to be ~58% more unstable than randomly selected genes [35]. This *Alu*-element based instability model is applied in the current study.

While closely spaced inverted DNA sequences are recognized as being unstable, the possible interaction of widely spaced repetitive elements is difficult to reconcile with known mechanisms for closely spaced inverted repetitive elements. Closely spaced inverted repetitive elements can interact by formation of single-stranded cruciform type motifs. However, the thermodynamic limitations associated with the formation of longer lengths of single-stranded DNA constrain the cruciform formation mechanism to closely-spaced, inverted sequences.

We are aware of only two proposed mechanisms that can account for the genome-wide depletion of over one million inverted *Alu* pairs [43]. These two mechanisms propose that interactions occur when coincident single-stranded motifs align between two inverted sequences. More specifically, the close three-dimensional proximity of replication forks and/or DNA breathing bubbles at inverted sequences can result in an ectopic DNA conformation. Cook *et al*. described this as a Doomsday Junction [35, 43]. Herein it is further hypothesized that Doomsday Junctions can occur between any two single-stranded reverse complements.

Recent work has shown that homologous *Alu* sequences of 15 bp or less in length can interact, even when separated by thousands of base pairs [44, 45]. Unfortunately, other repetitive sequences are much less prevalent than *Alu* elements and thus their interaction patterns cannot be as precisely modeled. The aim of the present study is to overcome the limitation of assessing the relative instability of less common reverse complement sequences by applying the algorithms developed from *Alu* element imbalances. Herein, we apply these *Alu*-element based instability algorithms to reverse complements of the most commonly encountered cancer-linked SVs seen within *EGFR* exon 19.

*EGFR* mutations are the most commonly occurring driver mutations in non-small cell lung cancer (NSCLC) with adenocarcinoma histology, which accounts for over 80% of all lung cancer. The mutation frequency of *EGFR* in adenocarcinoma of the lung within the United States is 20–30% and reaches almost 50% in patients from Asia-Pacific regions [46–48]. *EGFR* mutations have been well-characterized in lung cancer due to their relationship to clinical responses to *EGFR* tyrosine kinase inhibitors. Approximately 90% of all the activating mutations in the kinase domain of *EGFR* are deletions in exon 19 and point mutations of L858R in exon 21. For this reason, these mutations have been termed “classical” activating mutations [49]. *EGFR* exon 19 deletions are the most common driver mutations in adenocarcinoma of the lung, and among these, the most common deletion is an in-frame, 15 bp deletion that removes bases 52–66 from this 99 bp exon *(hg38 coordinates*, *chr7*:*55*,*174*,*773–55*,*174*,*787)*. This deletion removes five amino acid residues, (ELREA) 746–750, from the 1,210-residue *EGFR* protein. The 33 amino acids encoded by exon 19 of the *EGFR* gene extend from residues 729–761 [50]. Because this particular deletion is the most commonly seen in the *EGFR* gene, this deletion has been coined the “canonical deletion”. This canonical deletion is estimated to account for 62.5% of exon 19 damage linked to adenocarcinoma of the lung [50].

Herein, we utilize long-read sequencing to reconstruct the sequence of the individual haplotypes present within the EGFR landscape of two different lung cancer patients. This separation of diploid haplotypes is known as phasing. The phasing of haplotypes within cancer-linked regions of the genome provides the opportunity for examination of SV present within each haplotype. When genome sequencing read lengths exceed the frequency of SNVs (or other genome variants such as short indels or SVs) it becomes possible to separate diploid sequences into individual haplotypes. Long read sequencing thus greatly facilitates haplotype phasing and thereby provides the opportunity to examine genomic architecture of haplotypes that may be associated with cancer-linked mutations. Such examinations of the genome may provide clues to cancer etiology.

While many studies have examined phased cancer-linked landscapes, we are unaware of any that have examined putatively normal landscapes for their potential to generate driver mutations [51, 52]. Therefore, to our knowledge, this study is the first to use the genomic architecture within phased putatively normal landscapes to estimate the the relative potential of individual haplotypes to form a cancer-driver mutation.

## Results

### Instability estimates of overlapping 15 bp windows across *EGFR* exon 19

**Fig 1:**
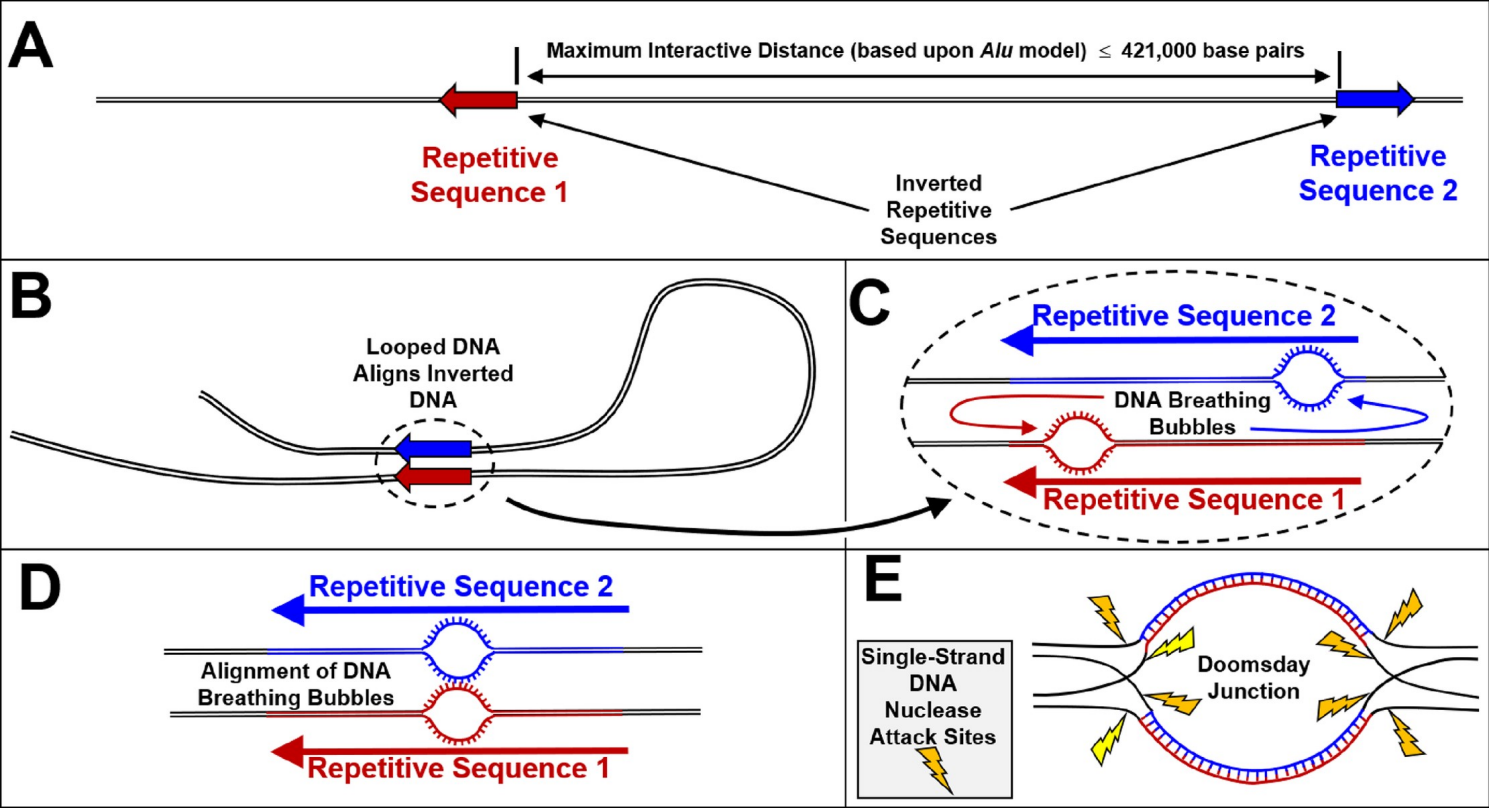
Proposed mechanism for the creation of double-strand DNA breaks resulting from the interaction of widely-spaced inverted repetitive sequences. A) Representation of an inverted repetitive sequence. Human inverted *Alu* element pairs are statistically depleted relative to direct-oriented *Alu* pairs up to a spacer size of 421,000 bp. B) DNA looping can create alignment of inverted pairs. C) Reactive single-stranded DNA is created both by DNA breathing bubbles as shown in this pane and also by replication forks. Note that the bases flip outward in breathing bubbles because of steric hindrance. This flipping out of bases makes the DNA more susceptible to interacting with a complementary breathing bubble. D) If single-stranded conformations of DNA occur in aligned inverted sequences, ectopic invasion and annealing can occur. E) Eight short stretches of DNA would be created as DNA makes the transition from its normal double-stranded form into a Doomsday Junction conformation. As with DNA hairpins, these short stretches of single-stranded DNA are susceptible to DNA nuclease attack (58). Random cleavage of the eight single-stranded DNA sites is hypothesized to continue until the Doomsday Junction is resolved. If both strands of the same DNA segment are cleaved, a double-strand break can be created.

**Fig 2:**
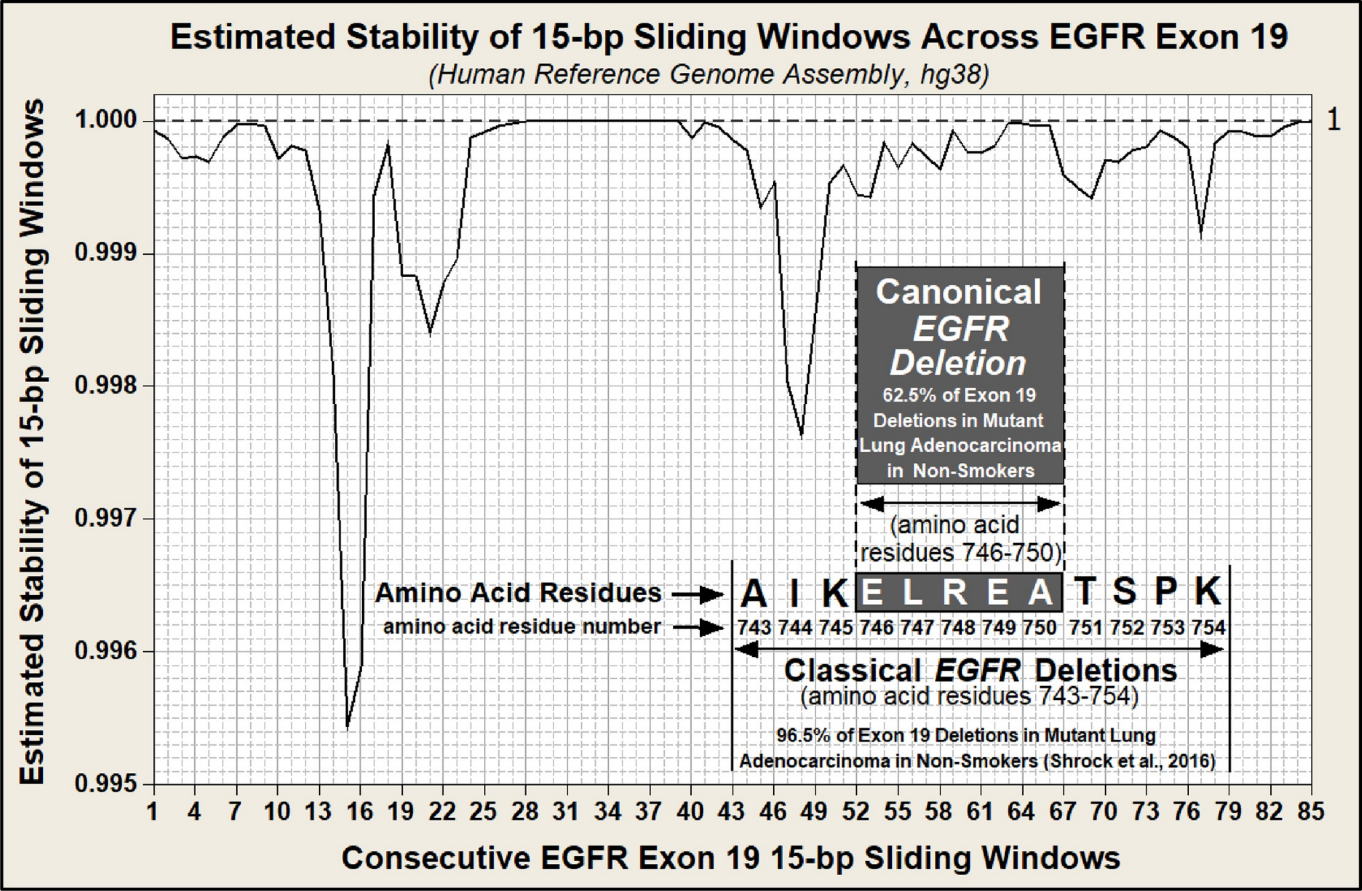
Estimated stability of overlapping 15 bp windows across *EGFR* Exon 19 of the hg38 reference genome assembly. Note that while this exon is comprised of 99 bp, the X axis covers only 85 bp. This is because the last 15 bp window extends from base number 85 through base number 99. The canonical exon 19, 15 bp deletion (amino acids ELREA) extends from nucleotides 52–66. Finally, the stability of this cancer-linked region lies within the top 20% of the most unstable 15 bp regions within exon 19. This finding suggests that some of these regions of higher instability may not create driver mutations when damaged.

**Fig 3:**
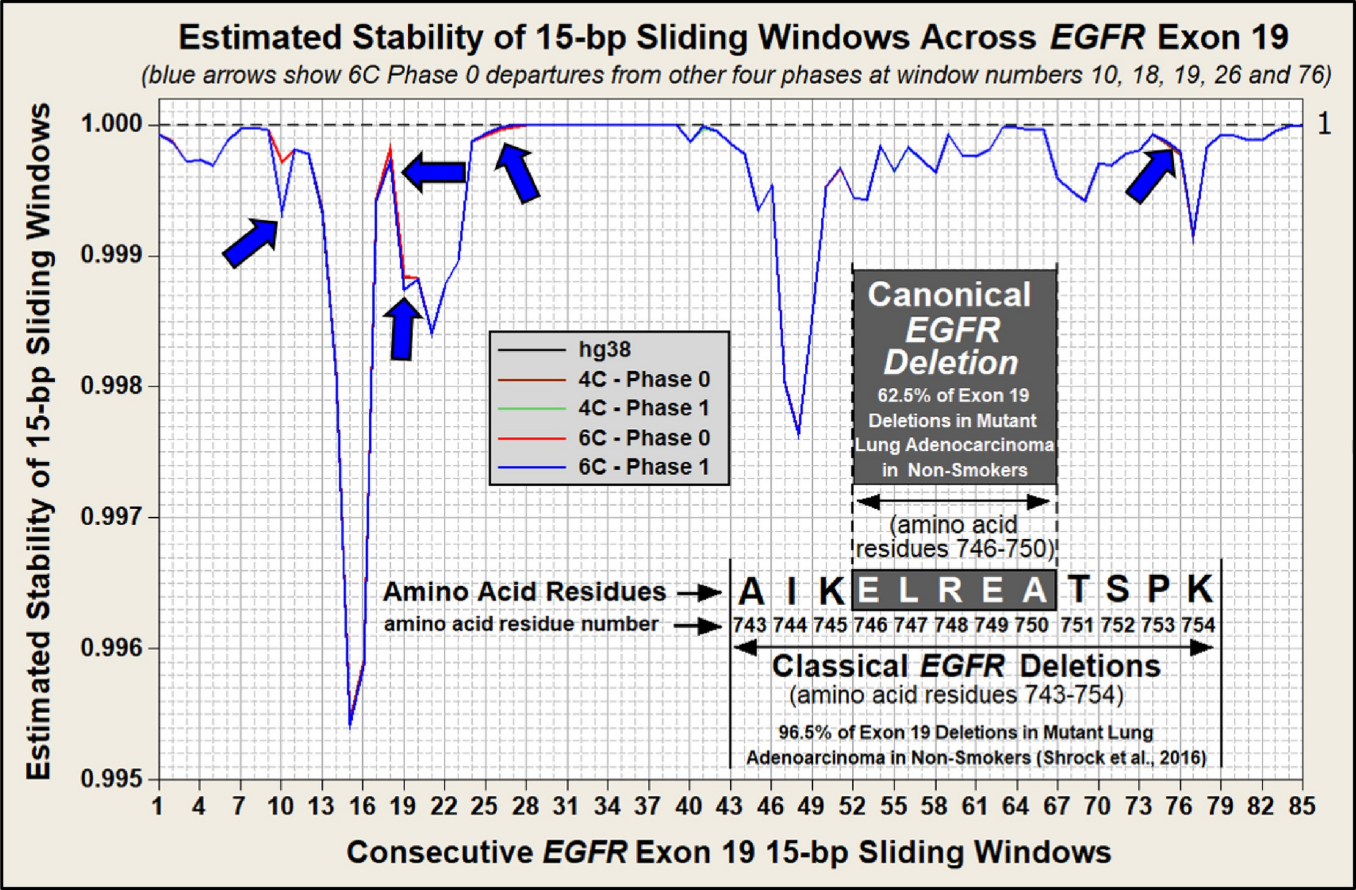
Estimated stabilities of *EGFR* exon 19 for five genomic phases with overlapping 15 bp windows. The increment for these windows is one base pair. Note that while these five phases present similar estimated stabilities, they are not identical. The blue arrows identify exon 19 regions where the stabilities are most dissimilar. As with Fig 2, the X axis covers only 85 bp. This is because the last 15 bp window extends from base number 85 through base number 99.

The hypothesized mechanism for the formation of a Doomsday Junction by closely aligned DNA breathing bubbles is described in Fig 1. Assuming the potential for widely spaced interactions between 15 bp reverse complements and using the *Alu* element-based instability model, we estimated genome instability for each 15 bp window within the coding sequence across *EGFR* exon 19 in the human reference genome (hg38), shown in Fig 2. Fig 3 is similar to Fig 2 but also includes our estimated *EGFR* exon 19 stabilities for each of the five haplotypes examined in this study. Note in both Fig 2 and Fig 3 that these 85 windows overlap in one base pair increments. This window size was chosen to match the size of the canonical deletion. Each window proceeds at a one base pair increment across the full length of this exon. Note that the last window begins at base 85 and extends through the last base in this 99 bp exon.

Excluding hg38, the four *EGFR* haplotypes described in Fig 3 are ostensibly stable genomic landscapes. These landscapes are considered “stable” because they have not generated an identified tumor with an *EGFR* exon 19 deletion in either of the two patients (four haploid genomes) used in this study. Note that while none of the four patient haplotypes resulted in an *EGFR* driver mutation, we were able to show that their genomic landscapes are not identical. Table 1 describes this haplotype-to-haplotype variation by both the number and distribution of reverse complements and by the calculated stabilities. These variations in estimated stabilities between the five different *EGFR* landscapes shown in Table 1 appear to be small as they occur after the fourth decimal place. However, when taken in the context of billions of cells in an organ, small changes in stability may result in measurable susceptibilities to a driver mutation. Small variations in landscape stabilities may be undetectable when presented in a chart such as the one shown in Fig 3. However, the blue arrows in Fig 3 denote visible variations in estimated stabilities between these haplotypes across all 85 of the 15 bp windows of *EGFR* exon 19.

**Table 1:** Reverse complement variation among five *EGFR* landscapes.

EGFR Landscapes^(^^1^^)^	No. of Reverse Complements by the No. of Mismatches^(^^2^^)^ within ± 421,000 base pair Landscapes						Raw Stability Score for Exon 19 Canonical Deletion Sequence^(^^3^^)^	Relative Stability	Raw Stability Score Excluding High Homology Reverse Complement^(^^4^^)^	Raw Stability Score Excluding High Homology Reverse Complement^(^^4^^)^
	2	3	4	5	6	Total				
**4C**	1	3	12	21	54	91	0.99944046	99.79%	0.999942454	97.90%
	1	3	11	20	54	89	0.99944165	100.00%	0.999943640	100.00%
**6C**	1	3	11	21	53	89	0.99944156	99.98%	0.999943553	99.85%
	1	3	12	19	55	90	0.99944063	99.82%	0.999942622	98.19%
**hg38**	1	3	11	20	55	90	0.99944161	99.99%	0.999943600	99.93%

**Fig 4:**
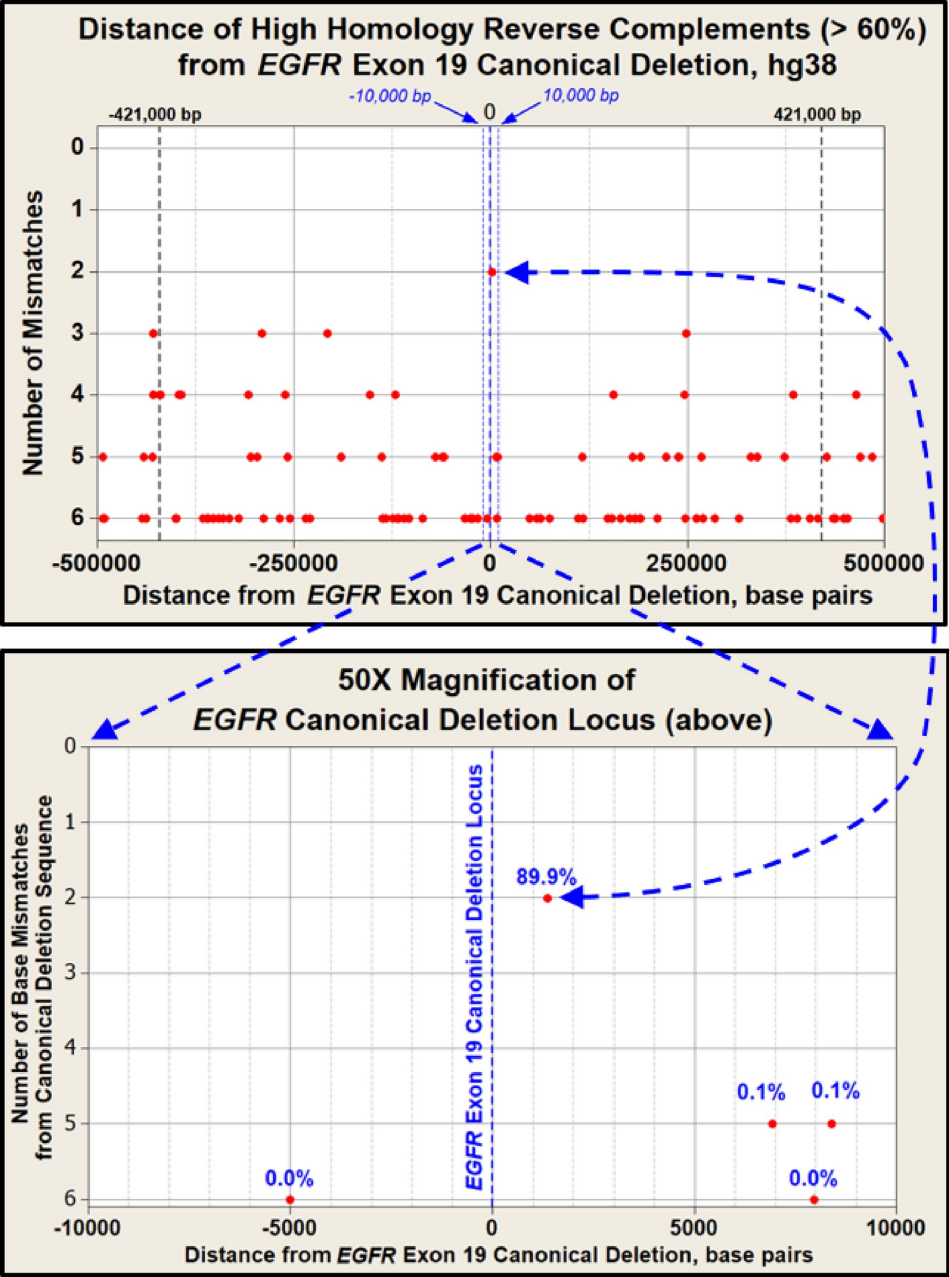
Loci of reverse complement sequences to the locus of the *EGFR* canonical exon 19 deletion (hg38). A) Total of 94 complementary sequences of 60% homology or greater are shown as red circles and fall within the reactive distance for inverted *Alu* element pairs. The Y axis is the number of mismatches found for each reverse complement located within the canonical deletion landscape. The maximum reactive distance of ± 421,000 bp is represented as black dashed lines [35]. Note that the most homologous locus to the canonical deletion is also the closest. This highly homologous locus accounts for almost 90% of the calculated instability of the exon 19 canonical deletion sequence. B) 50X magnified view of the canonical deletion landscape within ± 10,000 bp of the *EGFR* exon 19 canonical deletion locus.

(1) Phase designations within probands are arbitrary within patients because of phase discontinuities (see discussion).

(2) Number of mismatches compared to the *EGFR* exon 19 canonical deletion sequence.

(3) As compared to a ± 421,000 bp landscape with zero reverse complements to the *EGFR* exon 19 canonical deletion sequence (which has a raw stability score of 1.0).

(4) The total number of reverse complements among the five haplotypes ranges from 89–91. The highest homology reverse complement (Fig 4) contributes approximately 90% of the predicted reduction in stability for each haplotype. This largest contributor to reduced stability is identical for each haplotype and thus significantly reduces the variation in estimated stability between the phases. This final column highlights variation in remaining instability.

### Location of reverse complements to canonical exon 19 deletion

**Fig 5:**
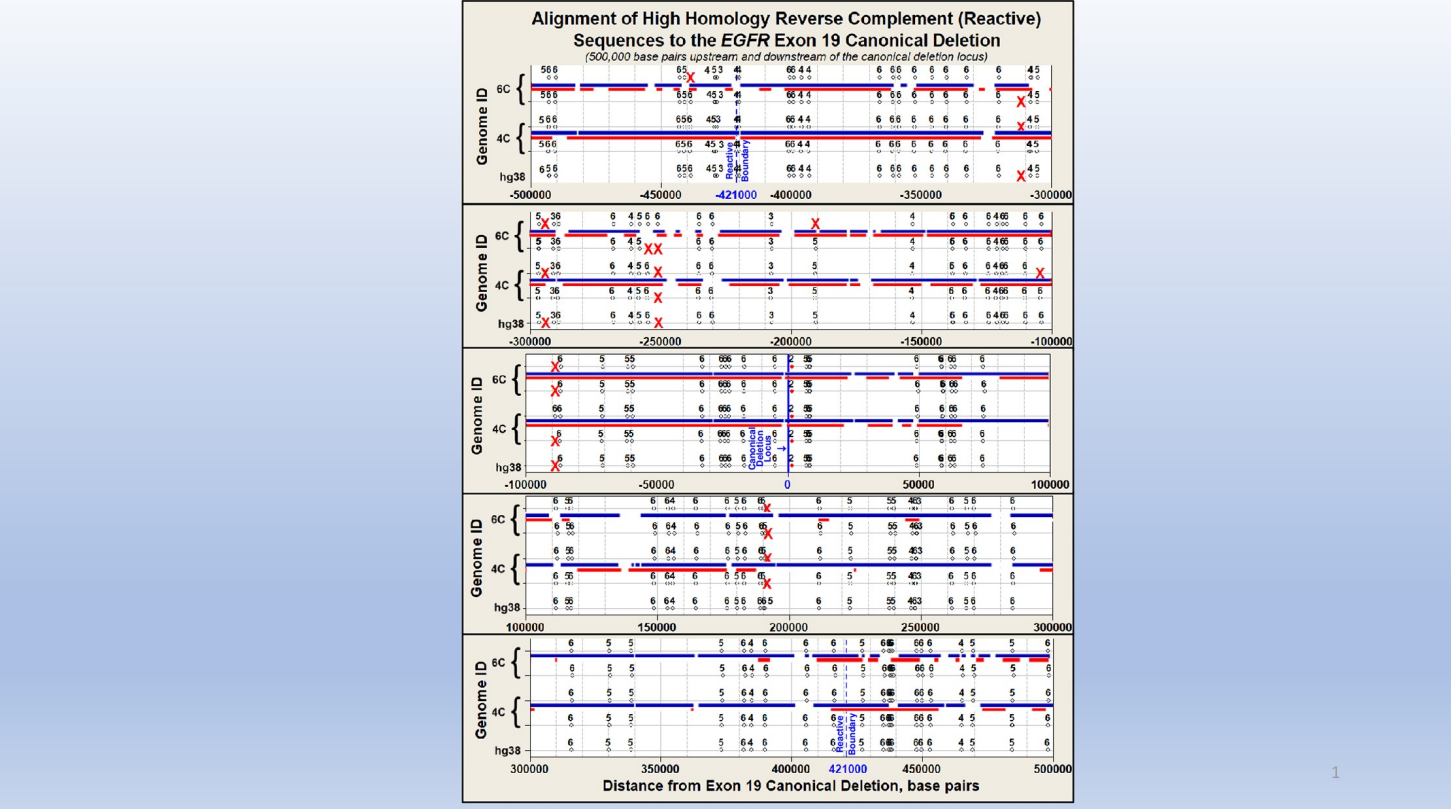
Reverse complement loci within ± 500,000 bp landscapes across five partially phased regions surrounding the *EGFR* exon 19 canonical deletion. This mega-base sized landscape spans from ± 500,000 bp of the *EGFR* exon 19 canonical deletion and is represented across five panes, each showing 200,000 bp. Although each of the five genome sequences in Fig 5 are identified on the Y-axis, the phase designations for each proband sequenced in this study are arbitrary as several low coverage phase discontinuities (see discussion) exist along each of these four landscapes. Horizontal red lines in each pane identify phased regions within each landscape. Horizontal blue lines identify regions where the depth of sequencing is 10-fold or greater. The absence of heterozygosity across some phase blocks prevented complete phasing of the landscapes. These regions of low heterozygosity (non-phased) are represented by blue lines only, without red lines. Note that the locus of the canonical deletion is located in the center-most pane at the X-axis value of “0” (blue line). Also note the very close proximity of the highest homology reverse complement to the canonical deletion locus which is designated as red circles. This reverse complement is separated by only 1,366 bp from the canonical deletion sequence. This is the closest locus among all 94 sets of reverse complements to the *EGFR* exon 19 canonical deletion sequence and are within the ± 421,000 bp region of reactivity flanking the canonical deletion. This region of reactivity is set by the statistical confidence of departure of the I:D ratio of *Alu* elements departure from unity (p<0.05) (41). These statistical confidence limits are shown as blue dashed lines at ± 421,000 bp from the canonical deletion locus. Except for the solid red circles denoting the high homology reverse complement, all remaining reverse complements are identified as black circles with the number of mismatches from the 15 bp canonical deletion shown above each circle. Each red X denotes a missing reverse complement that is present in one or more of the other landscapes. A total of 22 reverse complements are absent across one or more of these five haplotypes shown in this Fig 5. It is important to note that although phase identity is not possible within each proband, the presence of homologous phase blocks across the probands permitted identification of reverse complement variation across these five ± 500,000 bp landscapes.

Figs 4 and 5 illustrate all the respective loci we identified for all reverse complements to the *EGFR* exon 19 canonical deletion within 500,000 bp upstream and downstream of the *EGFR* exon 19 canonical deletion. These reverse complements are limited to 60 percent or greater homology with the 15 bp canonical deletion. That is, all SV having greater than six mismatches are excluded due to their much lower reactivity with the canonical deletion. Fig 4 provides these loci for the human reference genome (hg38) only and includes an expanded lower pane to show the very close proximity of the highest homology reverse complement to the canonical deletion locus in hg38.

Fig 5 describes the loci of all *EGFR* exon 19 canonical deletion reverse complements we identified for all five haplotypes evaluated in this study. These reverse complements are plotted across an area spanning 500,000 bp upstream to 500,000 bp downstream of the *EGFR* exon 19 canonical deletion and are illustrated in five separate 200,000 bp panes. Note that the locus of the canonical deletion is identified in the center-most pane at the X axis value of “0” (in blue).

We identified a total of 94 reverse complements across the five haplotypes with 60% or greater homology to the canonical deletion. Note that the highest homology reverse complement to the canonical deletion is also the closest in proximity to the canonical deletion locus at 1,366 bp from the canonical deletion locus. Using the *Alu*-based model of instability, this one reverse complement locus accounts for almost 90% of the total instability estimated for the canonical deletion locus. The region of reactivity between the canonical deletion and any flanking reverse complements is limited to the 421,000 bp upstream and downstream of the canonical deletion. This region of reactivity is set by the statistical confidence of the departure of the I:D ratio of *Alu* elements from unity (p<0.05) [35]. These statistical confidence limits are shown as dashed lines in Fig 5. Reverse complement loci are identified as circles with the number of mismatches with the canonical deletion shown above each circle.

**Fig 6:**
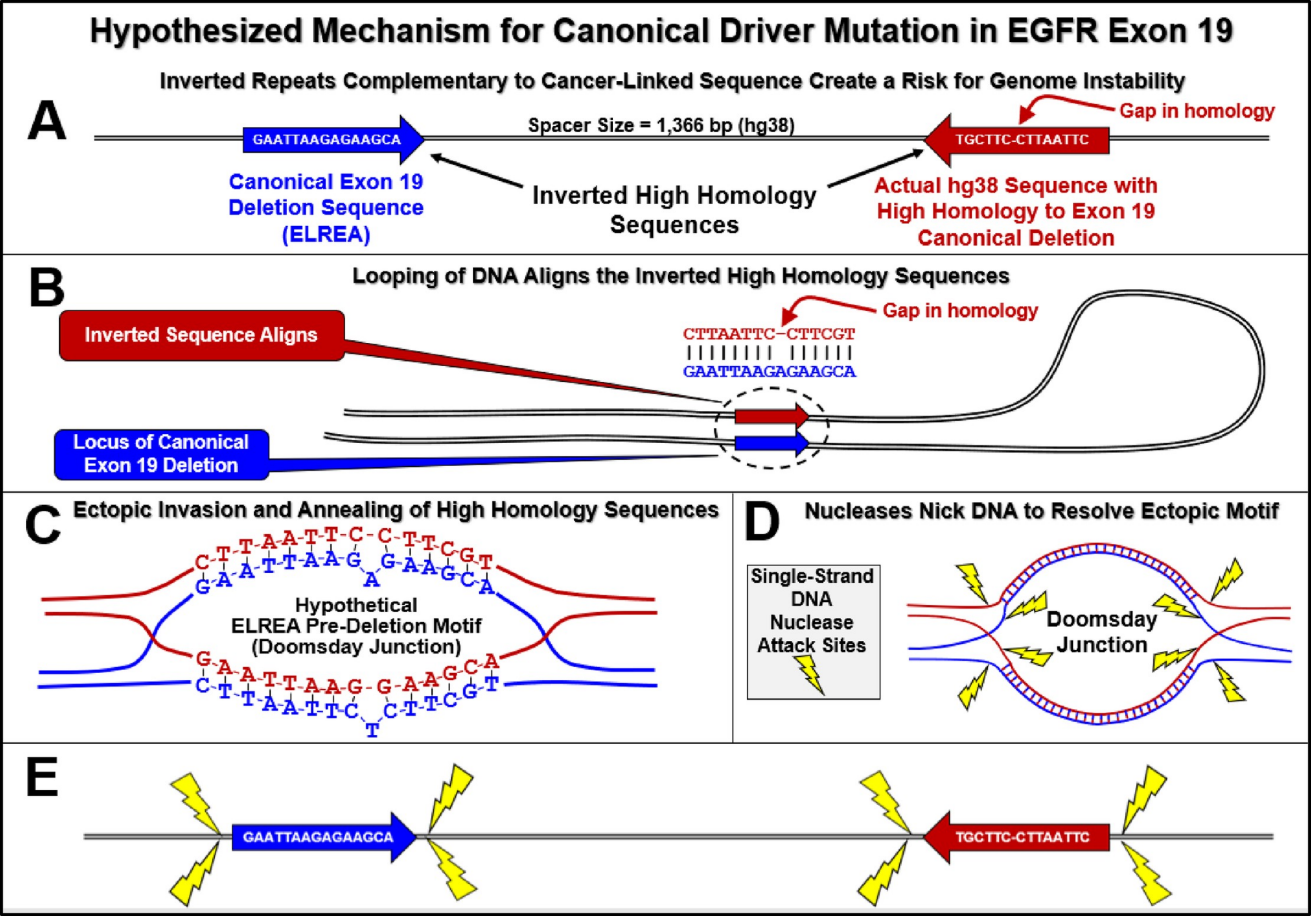
Hypothesized mechanism for canonical driver mutation in *EGFR* exon 19. A) Schematic of the region of both the *EGFR* exon 19 deletion prone region and its most homologous reverse complement. In this actual example from hg38, this reverse complement is located 1,366 bp 3’ from the *EGFR* exon 19 canonical deletion sequence. B) Alignment of these two sequences via formation of a 180° loop of DNA. C) Ectopic invasion and annealing (see also panes C, D, and E of Fig 1) through either aligned DNA breathing bubbles or replication forks. Note that the initial alignment is not perfect, and a single-base non-aligned region can occur D) Formation of Doomsday Junction. Putatively, this DNA conformation may be resolved through one or more nicks by single-strand nucleases at the transitional boundaries of this ectopic DNA conformation (see yellow lightning symbols). E) Regions of the inverted pair that are vulnerable to nicking. Comparing panes D and E, note that if a nick occurs in each of the two single-strands of DNA at the same end of a repetitive sequence, a double-strand break is created. If two single-stranded nicks occur at both ends of the exon 19 strand, the canonical deletion can be created.

Although there is only a one base mismatch between the closest reverse complement (1,366 bp downstream of the canonical deletion), it is counted as two mismatches because of the strained alignment structure in the pre-deletion ectopic conformation of the predicted Doomsday Junction (Fig 6, pane C). This gap mismatch penalty is imposed across all reverse complements identified in this study.

### The nearest reverse complement to the canonical deletion has highest homology

Table 1 summarizes the number of reverse complements to the canonical deletion that we identified among the five landscapes that are described in Fig 5. Table 1 and Fig 5 reveal that variation exists between normal repetitive landscape architectures. Table 1 provides evidence that individual genomes are not necessarily identical and raises the possibility that such variation may change cancer susceptibility among individuals. Fig 5 illustrates that most of the reverse complements to the canonical deletion are shared among the reactive regions of these five landscapes (± 421,000 bp). However, there are 21 instances where a reverse complement is missing from a specific haplotype and yet is present in one or more of the other four haplotypes. These loci are illustrated as red Xs. Application of the *Alu*-based instability model results in an estimated 0.2% variation in stability among the five haplotypes at the canonical deletion locus. This variation increases to 2.1% if the single high homology reverse complement described in Figs 4 and 6 is removed from the analysis (Table 1). While instability analysis was limited by the inability to create phased haplotypes across the entire 1 Mb locus, the variation among various sets of homologous reverse complements remains informative. Specifically, this “normal” human-to-human genomic variation among reverse complements to the *EGFR* exon 19 canonical deletion regions may result in measurable variation in cancer susceptibilities among individuals.

### In silico manipulations of the *EGFR* landscape

We examined the stability of the *EGFR* landscape via *in silico* manipulations when subjected to a 1,000 bp deletion, duplication, or inversion at the high homology reverse complement location. As expected, using our instability model, the hypothetical deletion and inversion reduced the instability by almost 90% while the hypothetical duplication almost doubled the instability. S1 Fig (supporting information) illustrates the impact of each of these hypothetical structural variants. The sensitivity of this reverse complement instability analysis was also performed using a hypothetical 20 bp reverse complement. While such a sequence would be approximately 1,000 times less likely to occur than the existing 15 bp reverse complement, this analysis validates the previously identified major region of instability (S2 Fig–supporting information).

## Discussion

The genomic architecture of a gene’s landscape can be complex. Genomic landscapes are not only characterized by the resident repetitive sequences, but also by the impact of SV which can alter repetitive sequence spacing, orientation, and homology. Consequently, while two people may share identical alleles for a given gene, the genomic stability of their respective DNA landscapes associated with their alleles may vary.

The advent of third generation long-read sequencing over the past decade allows us to now traverse segments of the genomic landscape previously hidden from view. Due to the longer fragments achievable by long-read sequencing, detection of large deletions, duplications, and complex rearrangements is now made possible [53]. In contrast to short-read technology, long-read technology is more likely to detect this structural variation with high-confidence alignments [5, 54]. Long read sequencing also provides for the possibility of haplotype resolution or “phasing” which allows for the assignment of genetic variants to the homologous maternal or paternal chromosome [55].

The *EGFR* gene contains 28 exons. Of these 28 exons, exon 19 is of particular interest in evaluation of structural variation and its potential impact on genome instability [56]. The exon 19 canonical deletion is only 15 bp but gives rise to one of the most common cancer-driver mutations in lung cancer. The exon 19 canonical deletion is one of the most common activating mutations in the never smoker lung cancer population, accounting for up to an estimated 25–30% of all *EGFR* activating mutations in lung cancer and approximately 60% of all exon 19 deletion damage in lung cancer [49, 50, 57–60]. This known “deletion prone” region covers a window that removes bases 52–66 from this 99 bp exon and five amino acid residues, (ELREA) 746–750, from the 1,210-residue *EGFR* protein. This region of the canonical deletion uniquely lends itself to study with the *Alu-*element instability model.

### Applying the *Alu*-element based model of human genome instability to the exon 19 canonical deletion reveals novel findings

The *EGFR* exon 19 canonical deletion possesses several characteristics that collectively provide a unique opportunity to assess the *Alu* element-based model of human genome instability [35]. These features make it an especially favorable mutation for study and are as follows:

1. This mutation occurs in cancer cells and therefore allows for study in clonally expanded tissues which provides sufficient genetic material for third generation sequencing.
2. The canonical *EGFR* exon 19 deletion is the most common cancer-linked driver mutation in lung cancer in never smokers. This relatively high frequency in the population of this mutation may facilitate validation of our proposed instability model.
3. This deletion fortuitously occurs entirely within the exon which allows for high confidence detection of its breakpoints with exome sequencing. Also, because coding regions of the genome are generally characterized by unique, non-repetitive sequence, identifying the reverse complement sequences to the canonical deletion sequence is less likely to be confounded by repetitive element sequences [61]. The absence of confounding repetitive sequences results in the likelihood of a random DNA sequence having ≥14 complementary matches within 1,366 bp of the15 bp *EGFR* exon 19 canonical deletion sequence of p<0.0003 (see Methods). Such a high homology sequence was identified in hg38 and is shown in Fig 6.

### Phase discontinuities do not prevent discovery of reverse complement variation

Four of the five *EGFR* landscapes shown in Fig 5 were obtained using PacBio long-read sequencing. The fifth landscape is the human reference genome assembly, hg38. Phase discontinuities within a genomic assembly can result from regions of low coverage or low heterozygosity. An average of 26 low coverage phase discontinuities (range of 18 to 30) are present within the ± 421,000 bp reactive landscapes within each of the four assembled long-read sequences. Haplotype assignment (i.e., haplotype 1, haplotype 2) of phased subsequences is consistent within each individual phased region, and not between phased regions. These assignments are illustrated in Fig 5.

The presence of these low coverage phase discontinuities prevents accurate phase identification within the respective genomic landscapes for each proband. However, it is important to note that reverse complement variation is observed across several sets of homologous phased sequences within the five *EGFR* genomic landscapes examined in this study. Adjusting for low coverage phase discontinuities, the total sequence coverage across these four landscapes is 97.6%.

This sequencing data provides accurate identification of 98.7% of the reverse complements across these four reactive landscapes (443 of 449). Horizontal red lines in each pane of Fig 5 identify phased regions within each landscape while horizontal blue lines identify regions where the depth of sequencing is 10-fold or greater. The absence of heterozygosity across some phase blocks prevented complete phasing of the landscapes. These regions of low heterozygosity (non-phased) are represented by blue lines only, without red lines.

It should be noted that the high homology reverse complement to the *EGFR* exon 19 canonical deletion resides within the same phase block as the canonical deletion sequence for all four PacBio sequenced landscapes (Fig 6).

A total of 94 different reverse complements (of ≥ 60% homology to the *EGFR* canonical deletion) were identified across the five *EGFR* ± 421,000 bp landscapes illustrated in Fig 5. A total of 21 of these reverse complements were absent across these five landscapes. The number of missing reverse complements were 8, 9 and 4 within the landscapes for 4C, 6C, and hg38, respectively. These sequence variations are consistent with the recent findings of hundreds of thousands of indels (<50 bp) and tens of thousands of SVs (>50 bp) within human genomes [1].

Finally, of the 449 total reverse complements identified across these five landscapes, six (1.3%) fell within low coverage phase discontinuities. The hg38 sequence was used to fill in the sequence across these low coverage regions, therefore, reverse complements that occur within them are those that are present in the hg38 sequence. The six reverse complements fell within coverage gaps once for 4C and five times for 6C.

### Not all cancer-linked gene mutations result in a cancer driver phenotype

Fig 2 predicts that the region of highest instability within *EGFR* exon 19 occurs in the first half (5’ end) of exon 19 within bases 13–24. If the *Alu* element-based instability model that was used to estimate the instabilities in Fig 2 is correct, this suggests that although DNA damage likely occurs more frequently in the 5’-end of the exon, only 3’-end damage generates the lung adenocarcinoma phenotype. Our instability model across *EGFR* exon 19 incorporates the observation by Schrock *et al*. that in 400 cases of lung adenocarcinoma linked to *EGFR* exon 19, all 400 deletions occurred in the latter half (3’ end) of this exon. The cancer-linked region of *EGFR* exon 19 driver deletions is overlaid on Fig 2 [50]. Note that within this cancer-linked region of exon 19, two windows containing bases 47–61 and 48–62 are more unstable than the canonical deletion. This may indicate that the canonical deletion creates a more aggressive driver mutation than deletions within these other two windows. Finally, note the 15 bp window containing the canonical deletion (bases 52–66) is within the top 20 percent of the most unstable windows across exon 19. These findings suggest that if cancer susceptibility is to be identified by an instability analysis of SV, that analysis must be guided by identification of cancer-prone regions of the genome.

Our results in this limited study of the genomic landscape surrounding the *EGFR* gene identify variation within the reverse complement architecture for the canonical driver mutation (exon 19 deletion) for this gene. Such variation has the potential to create “hot spots” for instability. This variation suggests that some individuals may be exposed to a greater susceptibility to the cancer-linked *EGFR* exon 19 canonical deletion. This genome-to-genome variation suggests a potential impact of SV upon cancer susceptibility among individuals.

## Conclusions

Using previously derived instability algorithms, coupled with long-read sequencing and phasing analyses, this work reveals the following new insights into the structural variation in the *EGFR* landscape and the associated estimated genomic instability.

1. The reverse complements (≥ 60% homology and within ± 421,000 bp) to the *EGFR* exon 19 canonical deletion varied across the five haploid genomes examined (4 patient landscapes and hg38). This finding suggests that there is normal human-to-human variation which may influence genomic instability.
2. Of the 116 reverse complements we identified across the ± 500,000 bp landscapes in Fig 5, the reverse complement with the highest homology (14 out of 15 bp = 93%) was also the closest to the canonical deletion at only 1,366 bp downstream (3’), which makes it more likely to interact with *EGFR* exon 19. This single locus accounts for almost 90% of the estimated genomic instability in the exon 19 canonical deletion, the most common cancer-linked mutation in the *EGFR* gene (Fig 6). The likelihood that such a highly homologous reverse complement would occur by random chance within this proximity to the exon 19 canonical deletion sequence (p<0.0003) is a compelling clue as to the possible mechanism for the formation of the exon 19 canonical deletion.
3. Although the sample size is limited in this study, the estimated variation observed in genomic stability between the five *EGFR* haplotypes examined is novel and encourages further work to examine structural variation in larger cohorts.
4. The estimated variation in *EGFR* stability may justify similar examinations of other disease-linked landscapes.

## Materials and methods

### Subjects and ethics statement

This study was approved by the Institutional Review Board of the University of Utah. The patients gave written consent under the Molecular Classifications of Cancer IRB #10924, which included tissue sampling from their lung cancer resection material, DNA extraction from tissues, and access to medical records. Two lung cancer cases were randomly selected from a larger cohort of non-squamous lung cancer surgical resection cases harvested from the Huntsman Cancer Hospital Thoracic Surgery service over an approximately two-year period. All tissue samples were procured and stored by the Huntsman Cancer Institute Biorepository and Molecular Pathology shared resource core. Both tumor and grossly uninvolved tissues were harvested in 30–40 mg aliquots. When available, two aliquots from tumor were flash frozen and two aliquots were fixed in PAXgene Tissue System (PreAnalytiX GmbH, Switzerland). The grossly uninvolved tissue was harvested and stored in a similar manner. All matching blood samples were collected in cell-free DNA blood collection tubes (Strek, La Vista, NE), centrifuged at 1900 rpm x 20 minutes and buffy coat extracted and stored at -80°C in cryovials. The two patients selected for study were originally selected as controls for protocol development and were selected without regard to mutational status. The sample utilized from patient 1 (labeled as 4C) was a buffy coat from the patient’s blood sample (germline) while the sample utilized from patient 2 (labeled as 6C) was from the patient’s tumor fixed in PAXgene formalin-free fixative and stabilizer, without paraffin-embedding. DNA was extracted from the blood sample using the Qiagen DNA extraction kit, per manufacturer’s instructions and from the PAXgene fixed tissue sample using the PAXgene DNA extraction kit, per manufacturer’s instructions.

### Long-read sequencing library preparation and custom targeted capture

DNA samples were fragmented to approximately 6 kb using a Covaris g-TUBE^™^. SeqCap® adapters (Roche) were ligated to 75–200 ng sheared DNA using the KAPA HyperPrep library preparation kit. Large insert library preparation included end repair, A-tailing, and adapter ligation. The sample libraries were amplified via ligation-mediated PCR (LM-PCR) using Takara Advantage® GC Genomic LA polymerase. After amplification, samples were size selected using a BluePippin instrument (Sage Science, Inc., Beverly MA, United States) with a size range of 3–10 kb. The fragment length distributions of the size selected libraries were assessed using an Agilent Bioanalyzer DNA 12000 chip. Universal blocking Oligos and COT Human DNA, 5 μl per reagent, from the HyperCap Target Enrichment kit (Roche) were added to 1 μg of amplified library for each hybridization reaction. Library plus reagents were concentrated using 2X AMPure XP reagent and then resuspended in 10μl 2X Hybe buffer and 4 μl Component A from the HyperCap Target Enrichment kit (Roche), 1 μl DMSO (Tedia High Purity Solvents, Fairfield OH, United States) and 5 μl biotinylated hybridization probes (Roche). Biotinylated hybridization probes were designed for four cancer-linked genes (*CDKN2A*, *CDKN2B*, *EGFR* and *STK11*) and 1 Mb of their respective surrounding landscapes, which included 500 kb upstream and 500 kb downstream of each targeted gene. Best efforts were made to tile across the entire region, while avoiding targeting highly repeated sequences. Where necessary, coverage calculations took advantage of the average library fragment size to skip over sequences that would result in high off-target capture. Hybridization was performed at 47°C overnight. Sample libraries bound to probes were captured after overnight hybridization for 45 minutes using 50 μl Dynabeads® M-270 Streptavidin (Life Technologies, Carlsbad CA, United States). The captured beads plus bead bound library were then washed following methods detailed in the SeqCap EZ Library SR User’s Guide v5.3. Exceptions to the washing procedures include hand mixing of samples in wash buffers instead of vortexing, hand mixing for ~10 seconds per wash and the samples were resuspended in 35 μl PCR grade water. Post-capture LM-PCR reactions were performed using Takara Advantage® GC Genomic LA polymerase with 17 total cycles in 100 μl total volume. In preparation for sequencing on a PacBio instrument, SMRTbell adapters were ligated onto the amplified post-capture samples.

### Sequence variant evaluation

Targeted capture of long DNA fragments were obtained from each sample and submitted for long-read sequencing (Pacific Biosciences, SMRT^®^ Sequencing with Sequel Binding Kit 2.0, Sequel Sequencing Plate 2.1).

After primary analysis, Circular Consensus Sequence (CCS) reads were generated using SMRT Analysis 6.0.0 for each dataset and aligned to the GRCh38 reference genome using minimap2 [62]. PCR duplicates from post-capture amplification were identified by mapping endpoints and tagged using a custom script (https://github.com/williamrowell/markdup). Short variants were joint-called using GATK4 HaplotypeCaller [63] and filtered by quality metrics (SNPs and >1bp indels, remove variants with QD<2; 1bp indels, remove variants with QD<5). The SNP sites that passed filtration were used in conjunction with the deduplicated CCS alignments for read-backed phasing with WhatsHap [64]. We generated consensus sequences by applying phased SNP information to the reference FastA sequence using the VCFtools package [65]. For each sample, the analysis results in regions with phased DNA haplotype landscapes (“haplotype separated”) separated by regions with one DNA landscape where the variants cannot be phased (“collapsed”).

### Statistical analysis

#### Likelihood of reverse complement matches to the EGFR exon 19 canonical deletion

Using a binomial analysis with a 25% chance of a match and a 75% chance of a mismatch, there are 32,768 possible match/mismatch combinations across a 15 bp sequence. Further, there are 31 possible sequences with ≥14 matches to the reverse complement of the exon 19 canonical deletion. These 31 combinations include 15 opportunities for a single mismatch, 15 opportunities for a single gap (the actual sequence shown in Fig 6) and one single combination for a perfect match. Therefore, the likelihood for any random 15 bp sequence to have this level of homology is p<0.0000001. The high homology sequence shown in Fig 6 is located 1,366 bp from the canonical deletion sequence. There are 2,734 opportunities (2 x (1366+1)) for such a sequence to occur. Thus the likelihood of a ≥14 bp reverse complement to be present within this distance from the canonical deletion is 2,734 x 0.0000001 (p<0.0003).

#### Inverted repeat relative reactivity vs homology

*Alu* elements typically share approximately 85% homology while MIR elements share approximately 70% homology [39, 66]. This lower homology between MIR elements likely explains why the ratio of inverted to direct MIR pairs is greater than 0.985, ±0.012, while the ratio of inverted to direct (I:D) *Alu* pairs is 0.955, ± 0.006, (p<0.05). S2 Table (supporting information) compares the ratio of inverted to direct *Alu* and MIR pairs with homology. This deviation in I:D versus homology is in general agreement with direct measurements of recombination rates between inverted *Alu* elements in genetically engineered yeast experiments. These experiments found that inverted *Alu* recombination rates drop by approximately an order of magnitude for each ten percent drop in homology [42]. S3 Fig (supporting information) is adapted from this original work. This dramatic drop in recombination rate, with decreasing inverted *Alu* homology, is consistent with the observed lower I:D deviation from unity in inverted human MIR elements discussed above. Using these two observations, the instability model used in this study does not consider inverted sequence homologies of less than 60% because such low homology sequences make a relatively insignificant contribution to genome instability as compared to that of higher homology sequences (S3 Fig). Therefore, reverse complements to the 15 bp *EGFR* exon 19 canonical deletion were limited to a maximum of 6 mismatches (60% homology). See Supporting Information for a further discussion of homology versus reactivity of complementary sequences.

#### Analysis of landscapes

The *EGFR* exon 19 canonical deletion is 15 bp in length. This deletion occurs entirely within this exon and does not extend into the intronic sequence. Consequently, even though exon 19 is 99 bp in length, only 85 sliding windows are possible within the exon without incorporating the intronic sequence. These 85 sequences are generated by moving the 15 bp window in one base pair increments from the 3’ end to the 5’ end across exon 19.

Figs 2 and 3 illustrate the estimated instability of each sequential 15 bp window across this exon. Figs 2 and 3 were constructed by first generating reverse complements to each of the 85, 15 bp windows. Reverse complements were generated using DNA Duster (https://users.soe.ucsc.edu/~kent/dnaDust/dnadust.html). Next, the locus, distance, and homology of each reverse complement across the ±421,000 bp reactive landscapes were identified. Only sequences with ≥ 60% homology to each 15 bp window were used in this analysis. Reverse complement loci were identified using the NCBI BLASTn online program (https://blast.ncbi.nlm.nih.gov/Blast.cgi?PROGRAM=blastn&PAGE_TYPE=BlastSearch&LINK_LOC=blasthome). These data were then input into the *Alu*-based algorithms to generate relative instabilities.

The reverse complement stabilities for each 15 bp window across *EGFR* exon 19 were calculated using algorithms generated from a previously reported *Alu*-element based instability model [35]. This model is derived from the imbalance in the ratio of inverted to direct-oriented *Alu* pairs that are present in the hg19 human genome assembly. This imbalance exists for *Alu* element pairs separated by up to 421,000 bp (<0.05). *Alu* elements are the most frequently repeated sequence in the human genome and thus provide the opportunity for rigorous statistical analysis. There are slightly over one million *Alu* elements present in the human genome and over 100 million *Alu* pairs within the ±421,000 bp of statistically detectible imbalance flanking each human *Alu* element. In construction of this model, *Alu* pairs were categorized into groups based upon various parameters, resulting in typical sample sizes of approximately 550,000. For this sample size and using binomial distribution analyses, inverted to direct *Alu* pair ratios below 0.995 are statistically significant (p<0.05). The ratio of adjacent human inverted to direct *Alu* pairs separated by 50–100,000 bp is 0.955 (S2 Table). Statistics and graphics were generated using Minitab 16.

## Supporting information

S1 File Discussion of homology versus reactivity of complementary sequences. (DOCX)

S1 Table Common human repetitive sequence. (TIF)

S2 Table Comparison of inverted to direct pair ratios of adjacent human Alu and MIR elements. (TIF)

S1 Fig Impact of different structural variants on EGFR Exon 19 stability. Impact on the estimated EGFR exon 19 stability of three different 1,000 base pair structural variants using 15 base pair sliding windows (hg38). These structural variants (deletion, inversion and duplication) occur at the same location (chr7:55,175,821–55,176,820) within the EGFR landscape. Each structural variant is one kilo base pair in size and located one kilo base pair downstream of exon 19 in intron 19. The exon 19 canonical deletion is identified in S1 Fig by the dashed green line and the loss of the five amino acid resides, ELREA. As with Figs 2 and 3, the increment for these overlapping windows is one base pair. Also note that while this exon is comprised of 99 bases, the X axis covers only 85 bases. This is because the last 15 base pair window extends from base number 85 through base number 99.
(TIF)

S2 Fig Predicted stability of 20-base pair sliding window across EGFR Exon 19. 20 bp sliding windows across *EGFR* exon 19 –This graph, because of the statistical likelihood of homology of 20 vs 15 bp windows) dampens the regions of instability identified in the 15 bp window graphs. However, the major regions of instability identified in the 15 bp windows graphs are still identified. Enlarging a window by 5 bp, reduces the likelihood of a perfect reverse complement by approximately a factor of 1,000. Note that S2 Fig, unlike the 15 bp window graphs, extends into introns 18 and 19 to capture all bases across exon 19. This was done to more accurately compare this graph to the 15 bp window instability graphs. The large windows used to generate this graph would have reduced of the number of bases covered across exon 19 and made the two-graph comparison less informative.
(TIF)

S3 Fig Inverted Alu element pair recombination rate vs. homology. Relative reactivity of inverted Alu elements vs Homology–adapted from (8). Note that the interaction between inverted Alu elements drops approximately one order of magnitude for each 10 percent drop in homology.
(TIF)
