# Supplementary material for: Structural variation and its potential impact on genome instability: Novel discoveries in the EGFR landscape by long-read sequencing
Source: PLoS One. 2020 Jan 15;15(1):e0226340. doi: 10.1371/journal.pone.0226340 (PMC6961855; doi:10.1371/journal.pone.0226340)
Supplement: S1 File — (DOCX) [file pone.0226340.s001.docx]

**Supporting Information**

**Interactions between repetitive DNA elements**

Repetitive sequences have been estimated to make up approximately two-thirds of the human genome [1]. An overview of the most common human repetitive sequences is provided in S1 Table. The majority of the repetitive sequence in the human genome is the result of transposable element insertion events. While insertion events into genes have been shown to result in multiple disease states, researchers have noted that subsequent interactions between these highly homologous sequences may pose a more pronounced threat to human health than their insertions [2, 3].

Inverted DNA sequences have long been recognized as unstable motifs. However, beyond the formation of cruciform/hairpin structures, we are aware of only two mechanisms that have been described for how more widely spaced inverted DNA sequences might interact. These widely-spaced interactions (>100 bp), are hypothesized to occur by the formation an ectopic DNA structure know as a Doomsday Junction. Doomsday Junctions could potentially result from the invasion and annealing of inverted single-stranded sequences between replication forks and/or DNA breathing bubbles. This latter conformation is described in Fig 1 as a Doomsday Junction.

The hypothesized formation and subsequent resolution of Doomsday junctions provide the only explanation of which we are aware for the exclusion of over one-million inverted *Alu* pairs (as compared to direct-oriented *Alu* pairs) in the human genome [4, 5]. The depression in inverted *Alu* pairs as compared to direct-oriented *Alu* pairs is approximately 10-15% for *Alu* pairs separated by ≤ 5,000 base pairs and between 5-10% for separations of 5,000 - 40,000 base pairs [5]. This study extends the Doomsday Junction model for interactions between inverted *Alu* elements to the interaction of more rare inverted DNA sequences such as the *EGFR* canonical exon 19 deletion sequence and its high homology reverse complement described in Fig 6.

**Repetitive sequence homologies inversely correlate with I:D ratio**

*Alu* elements typically share approximately 85% homology while MIR elements share approximately 70% homology [6, 7]. This deviation in I:D versus homology is in general agreement with direct measurements of recombination rates between inverted *Alu* elements in genetically engineered yeast experiments. These experiments found that inverted *Alu* recombination rates drop by approximately an order of magnitude for each ten percent drop in homology [8]. S3 Fig is adapted from this original work. This drop in recombination rate, with decreasing inverted *Alu* homology, is consistent with the observed lower I:D deviation from unity in inverted human MIR elements discussed above. Using these two observations, the instability model used in this study does not consider inverted sequence homologies of less than 60% as lower homology interactions should make an insignificant contribution to genome instability (S3 Fig). Therefore, inverse complements to the 15 base pair *EGFR* exon 19 canonical deletion were limited to a maximum of 6 mismatches (60% homology).

**Motifs and Frequencies of EGFR Exon 19 Driver Mutations**

Most, if not all, tumors containing *EGFR* exon 19 damage are in-frame deletions [9]. The paucity of out-of-frame repairs at this locus suggests that the *EGFR* driver protein requires in-frame DNA repair. Furthermore, many cells with the canonical deletion may not obtain the necessary complement of necessary mutations to acquire the cancer phenotype [10, 11]. Additionally, other occurrences of the canonical deletion may have acquired the phenotype, but not have clonally expanded to the point where a tumor is detected before death occurs by other causes.

**S2 Table**

**References**

1. de Koning AJ, Gu W, Castoe TA, Batzer MA, Pollock DD. Repetitive elements may comprise over two-thirds of the human genome. PLoS Genetics. 2011;7(12):e1002384.

2. Deininger PL, Batzer MA. Alu repeats and human disease. Molecular Genetics Metabolism. 1999;67(3):183-93.

3. Hedges D, Deininger P. Inviting instability: transposable elements, double-strand breaks, and the maintenance of genome integrity. Mutation Research/Fundamental Molecular Mechanisms of Mutagenesis. 2007;616(1):46-59.

4. Cook GW, Konkel MK, Major JD, Walker JA, Han K, Batzer MA. Alu pair exclusions in the human genome. Mobile DNA. 2011;2(1):10.

5. Cook GW, Konkel MK, Walker JA, Bourgeois MG, Fullerton ML, Fussell JT, et al. A comparison of 100 human genes using an alu element-based instability model. PLoS One. 2013;8(6):e65188.

6. Smit AF, Riggs AD. MIRs are classic, tRNA-derived SINEs that amplified before the mammalian radiation. Nucleic Acids Research. 1995;23(1):98-102.

7. Stenger JE, Lobachev KS, Gordenin D, Darden TA, Jurka J, Resnick MA. Biased distribution of inverted and direct Alus in the human genome: implications for insertion, exclusion, and genome stability. Genome Research. 2001;11(1):12-27.

8. Lobachev KS, Stenger JE, Kozyreva OG, Jurka J, Gordenin DA, Resnick MA. Inverted Alu repeats unstable in yeast are excluded from the human genome. The EMBO Journal. 2000;19(14):3822-30.

9. Schrock AB, Frampton GM, Herndon D, Greenbowe J, Wang K, Lipson D, et al. Comprehensive genomic profiling identifies frequent drug sensitive EGFR exon 19 deletions in NSCLC not identified by prior molecular testing. Clinical Cancer Research. 2016:clincanres. 1668.2015.

10. Hanahan D, Weinberg RA. The hallmarks of cancer. Cell. 2000;100(1):57-70.

11. Hanahan D, Weinberg RA. Hallmarks of cancer: the next generation. Cell. 2011;144(5):646-74.

12. Mandal PK, Kazazian HH. SnapShot: vertebrate transposons. Cell. 2008;135(1):192-. e1.

13. Batzer MA, Deininger PL. Alu repeats and human genomic diversity. Nature Reviews Genetics. 2002;3:370. doi: 10.1038/nrg798.
