# Supplementary figures and images for: Structural variation and its potential impact on genome instability: Novel discoveries in the EGFR landscape by long-read sequencing
Source: PLoS One. 2020 Jan 15;15(1):e0226340. doi: 10.1371/journal.pone.0226340 (PMC6961855; doi:10.1371/journal.pone.0226340)

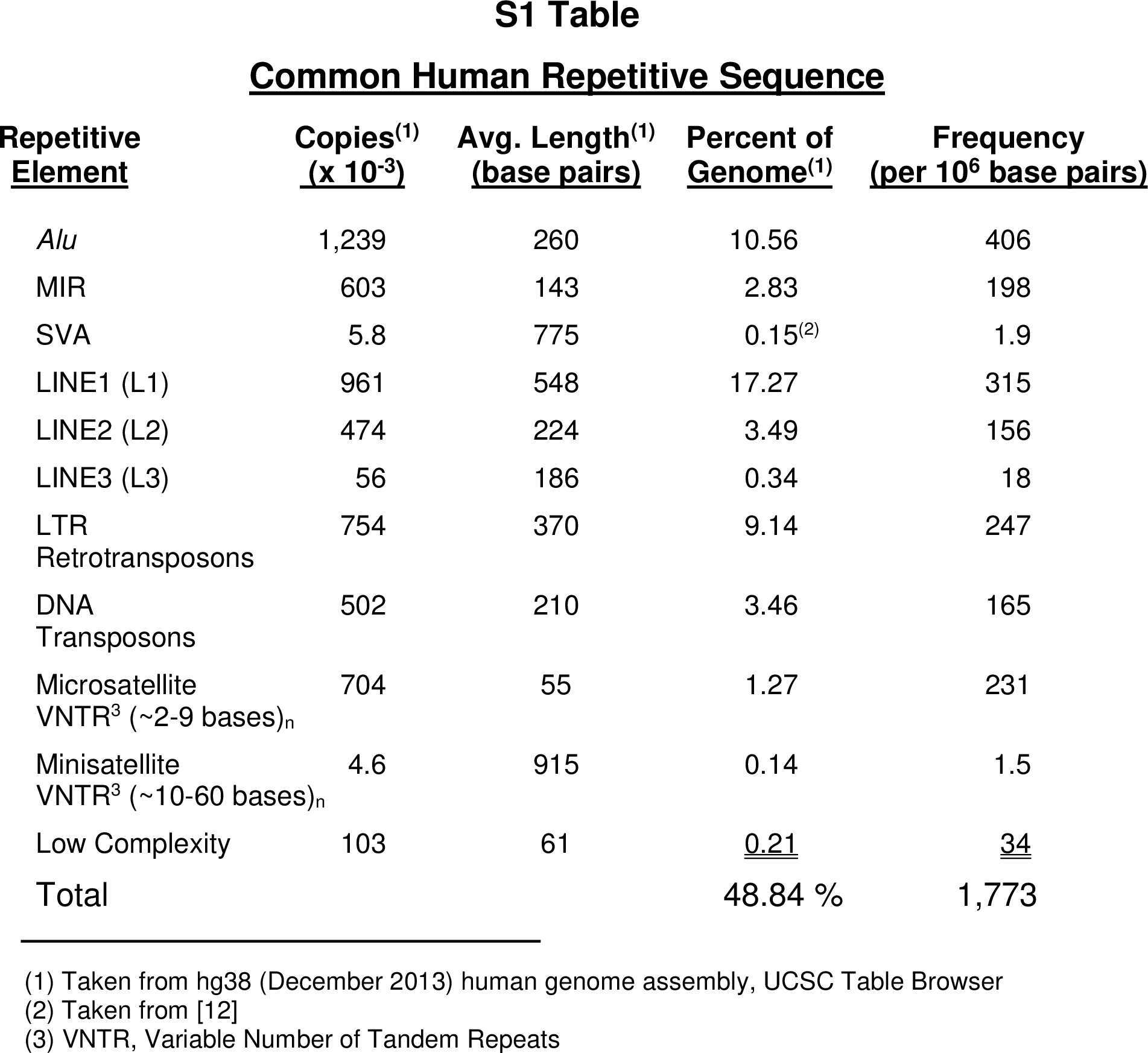

Supplement: S1 Table — (TIF) [file pone.0226340.s002.tif]

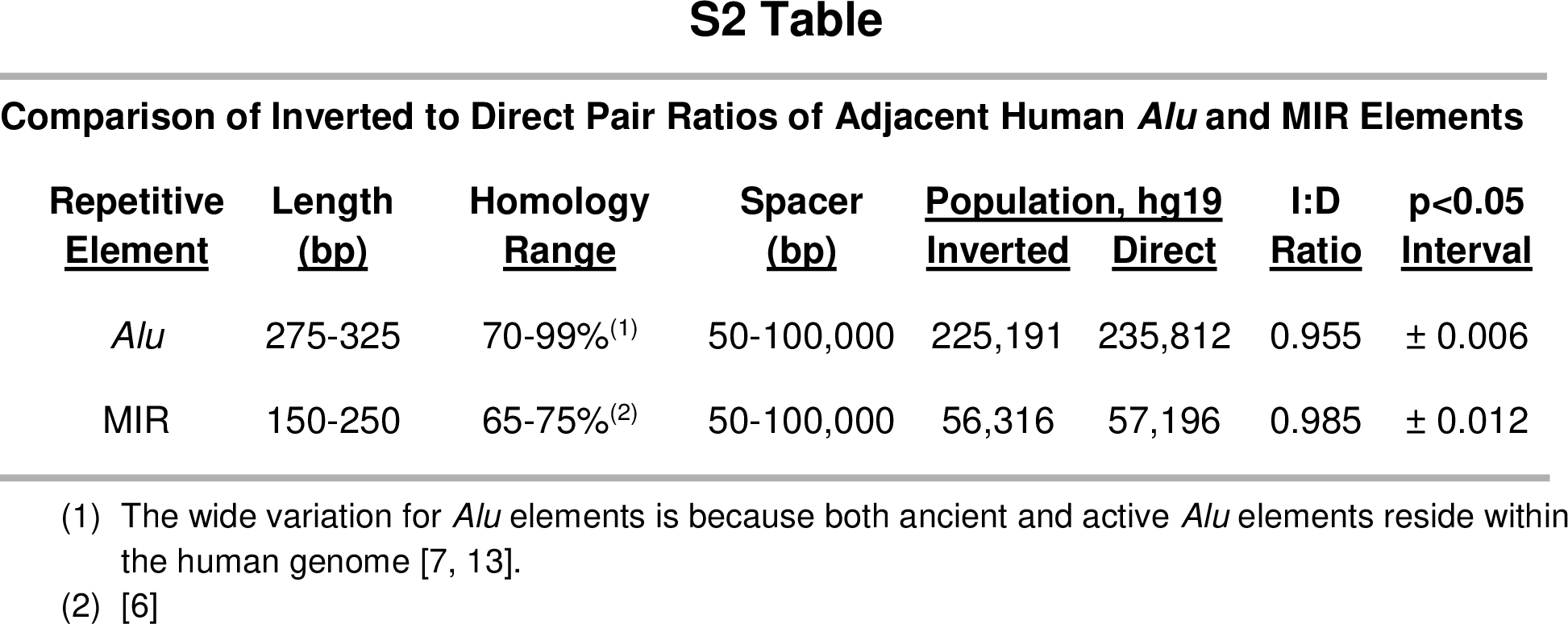

Supplement: S2 Table — (TIF) [file pone.0226340.s003.tif]

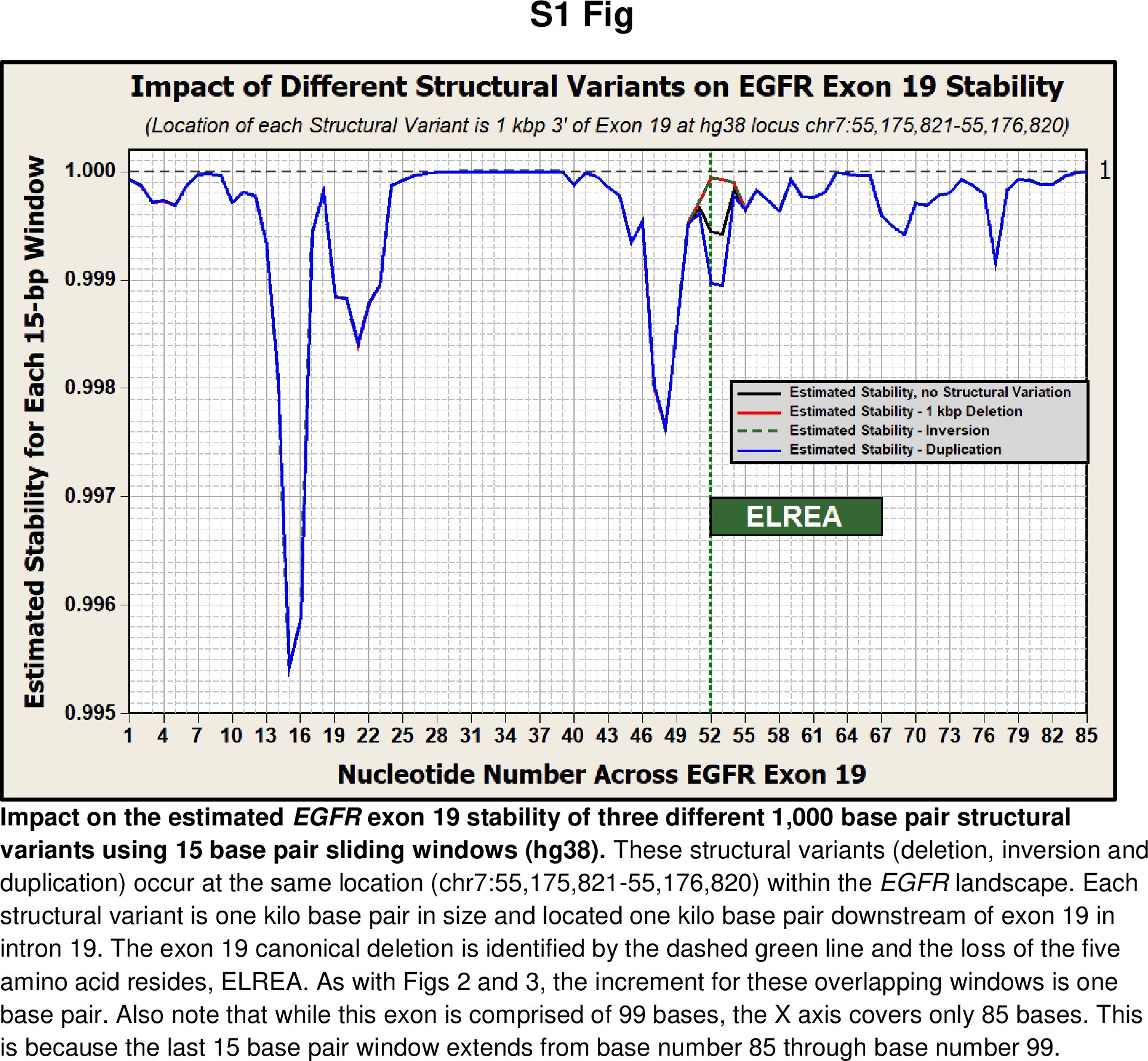

Supplement: S1 Fig — Impact on the estimated EGFR exon 19 stability of three different 1,000 base pair structural variants using 15 base pair sliding windows (hg38). These structural variants (deletion, inversion and duplication) occur at the same location (chr7:55,175,821–55,176,820) within the EGFR landscape. Each structural variant is one kilo base pair in size and located one kilo base pair downstream of exon 19 in intron 19. The exon 19 canonical deletion is identified in S1 Fig by the dashed green line and the loss of the five amino acid resides, ELREA. As with Figs 2 and 3, the increment for these overlapping windows is one base pair. Also note that while this exon is comprised of 99 bases, the X axis covers only 85 bases. This is because the last 15 base pair window extends from base number 85 through base number 99. (TIF) [file pone.0226340.s004.tif]

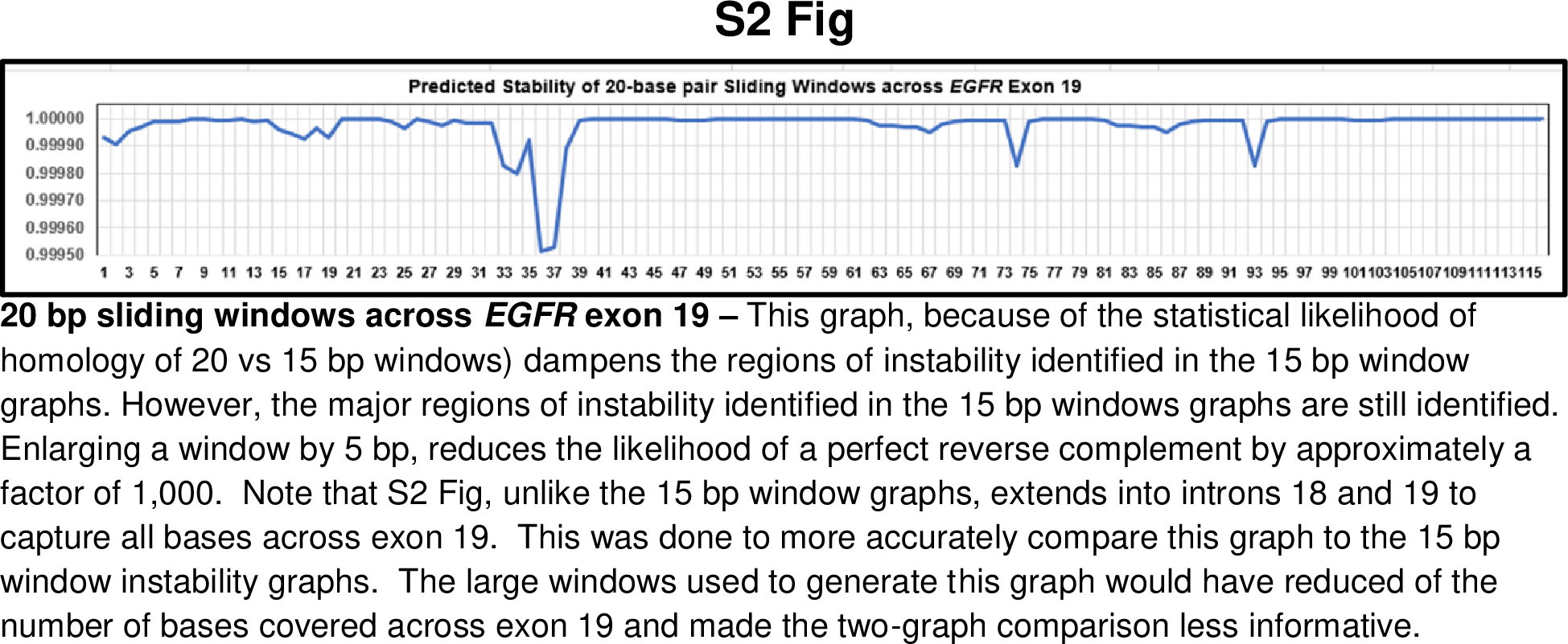

Supplement: S2 Fig — 20 bp sliding windows across EGFR exon 19 –This graph, because of the statistical likelihood of homology of 20 vs 15 bp windows) dampens the regions of instability identified in the 15 bp window graphs. However, the major regions of instability identified in the 15 bp windows graphs are still identified. Enlarging a window by 5 bp, reduces the likelihood of a perfect reverse complement by approximately a factor of 1,000. Note that S2 Fig, unlike the 15 bp window graphs, extends into introns 18 and 19 to capture all bases across exon 19. This was done to more accurately compare this graph to the 15 bp window instability graphs. The large windows used to generate this graph would have reduced of the number of bases covered across exon 19 and made the two-graph comparison less informative. (TIF) [file pone.0226340.s005.tif]

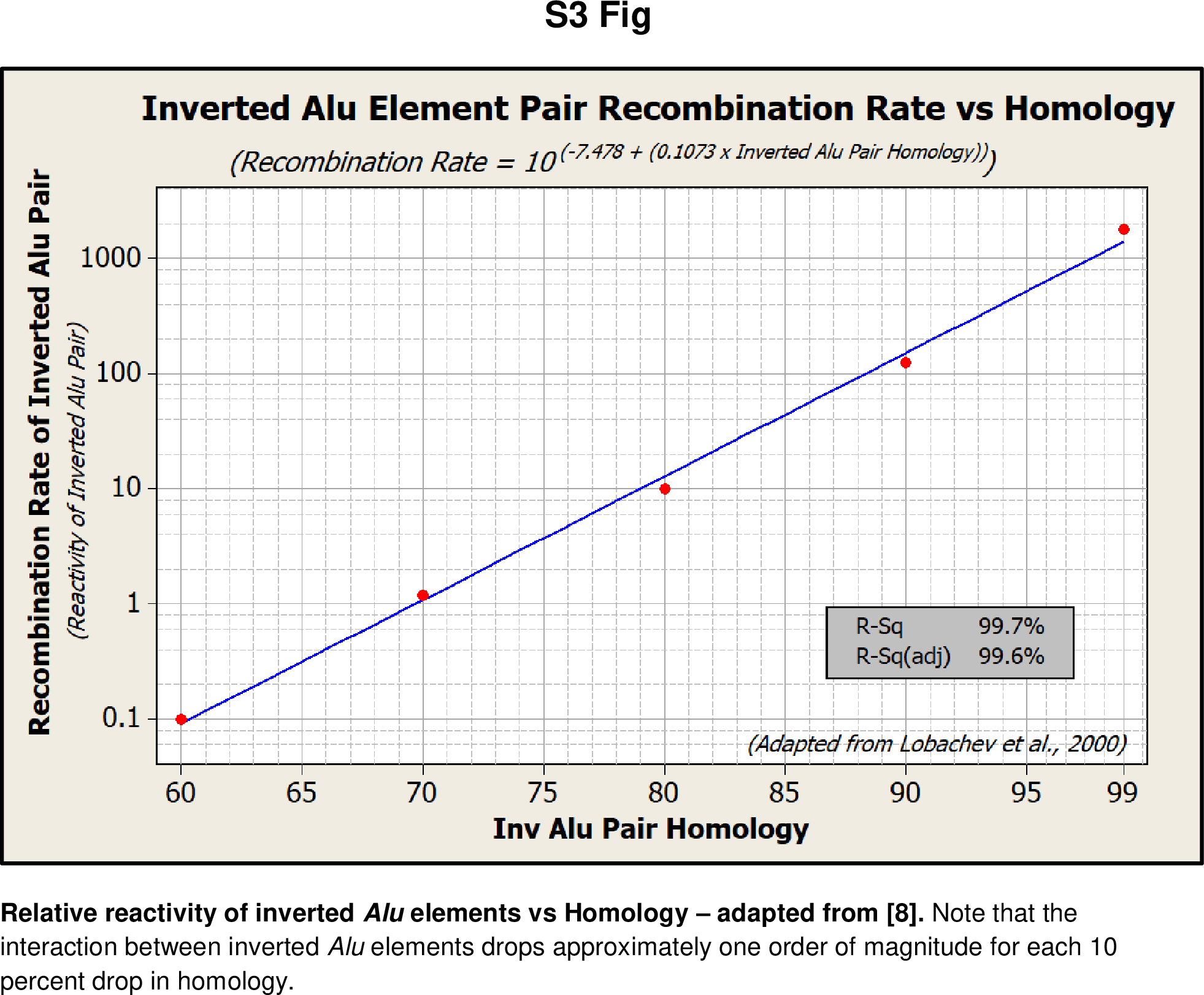

Supplement: S3 Fig — Relative reactivity of inverted Alu elements vs Homology–adapted from (8). Note that the interaction between inverted Alu elements drops approximately one order of magnitude for each 10 percent drop in homology. (TIF) [file pone.0226340.s006.tif]
